# Case study in VersKiK: a methodological approach for studying paediatric cancer survivors’ pathways

**DOI:** 10.1186/s12874-025-02723-x

**Published:** 2025-12-02

**Authors:** E. Aleshchenko, T. Langer, G. Calaminus, J. Glogner, J. Gebauer, E. Swart, K. Baust

**Affiliations:** 1 Institute of Social Medicine and Health Systems Research, Faculty of Medicine, Otto von Guericke University, Magdeburg, Germany; 2 University Hospital of Schleswig-Holstein, Campus Lübeck, Lübeck, Germany; 3 Department of Pediatric Hematology and Oncology, University Hospital Bonn, Bonn, Germany

**Keywords:** Pediatric cancer, Cancer survivorship, Long-term follow-up care, Late effects, Follow-up guidelines, Follow-up pathways, Transition to adult care, Case study

## Abstract

**Background:**

Advancements in medical treatment have significantly increased the likelihood of survival after childhood and adolescent cancer. However, this expanding group remains vulnerable to various late effects resulting from cancer itself or cancer treatment. It is crucial to implement consistent and systematic follow-up care procedures to promptly identify and address potential complications that may arise later in life.

**Methods:**

We conducted 19 unstructured participant observations of follow-up appointments and 36 episodic narrative interviews with paediatric cancer survivors (diagnosed before age 18) and their informal caregivers. We analysed observational field notes and personal narratives on the “survivor pathway” from interview transcripts, applying the inductive narrative method to Yin’s approach to case study development. Synthesising frequently discussed topics, we generated case studies to discuss with healthcare professionals and patient representatives in a focus group setting.

**Results:**

We designed two case studies to capture the complexity of follow-up care organisation in paediatric cancer survivorship for further discussion in focus groups with healthcare professionals. One case study describes a typical ‘survivor pathway’ of an adult survivor of paediatric cancer, and another describes a survivor currently transitioning from paediatric to adult healthcare facilities.

**Conclusions:**

Our objective is to examine real-life survivorship scenarios with the overall aim of suggesting improvements to the current structure of paediatric cancer follow-up care in the framework of a larger VersKiK-Study. We used both case studies as a basis for discussion in four focus groups (ca. 8 participants each) with healthcare providers involved in paediatric cancer follow-up and patient advocates.

**Supplementary Information:**

The online version contains supplementary material available at 10.1186/s12874-025-02723-x.

## Background

A significant proportion of paediatric cancer survivors suffer from lifelong late effects caused by cancer or its therapy and require continuous follow-up care [1, 2], resulting in special organisational and psychosocial needs. While current research typically focuses either on individual late effects or on specific health conditions they cause (e.g [3, 4])., or provides general overviews of survivors’ follow-up engagement (e.g [5]). Survivors experience multiple interconnected effects across all domains of their lives [6]. Survivors encounter additional difficulties during the transition from pediatric to adult healthcare facilities. This transition represents a complex process for adolescents who have received cancer treatment, encompassing developmental, health-related, and organisational changes [7]. Already navigating a vulnerable developmental phase characterised by significant physical, emotional, cognitive, and social transformations [8], these young people face the added challenges of unfamiliar healthcare environments and unknown medical professionals, while being expected to independently manage organisational responsibilities. Psychosocial challenges typical for this age, such as progress toward autonomy, gaining independence from their parents, developing a sense of identity, exploring intimacy, completing their education, and initiating their professional careers and family formation, represent an additional burden and may conflict with cancer late effects and the necessity of adequate care [9, 10].

Moreover, family members are significantly affected by survivors’ experiences and often face the necessity of providing multidimensional support, including physical, emotional, social, and financial assistance, to meet the complex needs of former cancer patients [11]. The presence of an informal caregiver is shown to generally enhance coping abilities and the adoption of healthier lifestyle habits among cancer patients [12]. Nevertheless, a significant number of caregivers feel unprepared to manage the challenges associated with follow-up care for their children [13, 14]. Prior research has underscored the importance of comprehending the unmet needs of informal caregivers for cancer survivors and formulating sufficient strategies to enhance their well-being, thus supporting survivors themselves [15, 16].

To support paediatric cancer survivors and their informal caregivers, national and international long-term follow-up (LTFU) guidelines were developed [17–19], and specialised centres were created [20]. In Germany, a limited number of survivors benefit from specialised LTFU care, due to constrained LTFU provider capabilities [20, 21]. Therefore, the majority of them have to organise and manage their follow-up care by themselves. The lack of guidance through follow-up care results in unique “survivor pathways” and might reduce adherence to the follow-up recommendations [21–23].

The present study aims to reflect survivors’ experiences of follow-up care organisation in the form of case studies, i.e. typical “survivor pathways”, to be further discussed in focus groups. The case study method, described as “an empirical investigation exploring a current phenomenon within its real-world context” [24], offers a chance to examine unique behaviours as both a component and a response to the surrounding conditions [25]. Unlike thematic analysis or phenomenological studies that focus on individual experiences, Yin’s case study methodology allows for the systematic integration of multiple data sources (such as observations and narratives) to create comprehensive, bounded cases that capture the complexity of survivorship pathways within the real-world context. This approach is particularly valuable for survivorship research as it enables the examination of cause-and-effect relationships and contextual factors that influence healthcare experiences, lacking in individual interview studies.

This study is a part of the larger VersKiK project. The mixed-methods VersKiK study was developed to provide a comprehensive basis for evaluating the long-term effects of childhood and adolescent cancer in Germany [26–28]. It includes quantitative analyses of anonymously linked Childhood Cancer Registry and statutory health insurance data to quantify the late effects of paediatric cancer, additional analyses of treatment data for several diagnoses to evaluate the adherence to follow-up guidelines and a qualitative module to study actual needs and views on follow-up care of paediatric cancer survivors, their informal caregivers and healthcare providers. The case study development represents one component of the larger project’s qualitative strand, designed to inform subsequent focus group discussions with healthcare professionals and patient advocates.

## Methods

### Design

**Fig. 1:**
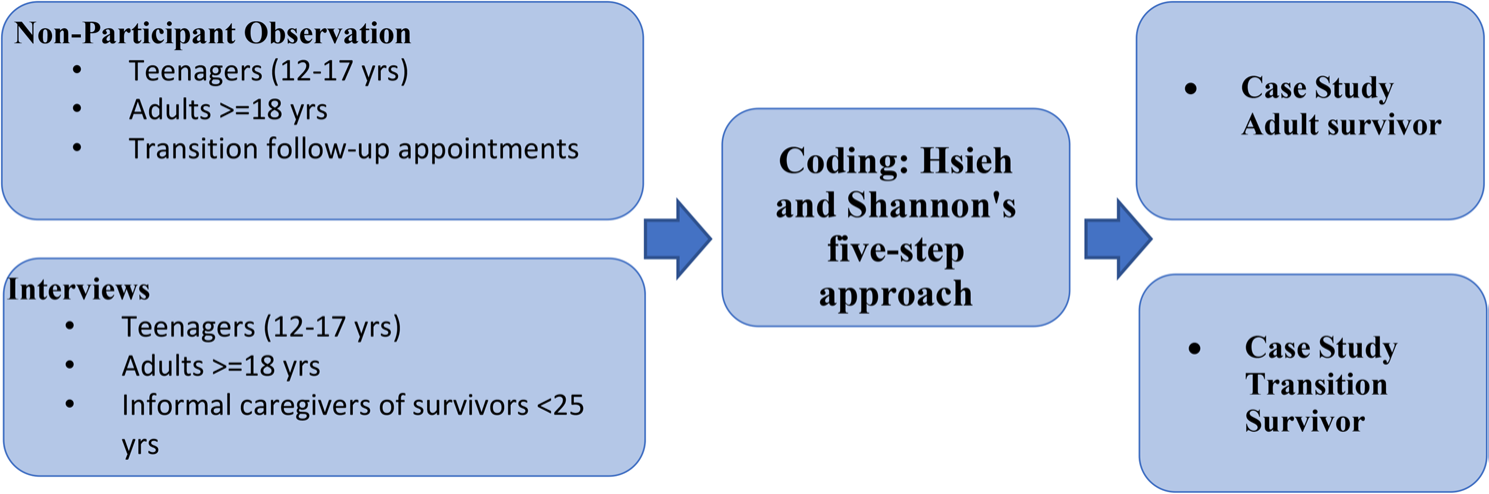
Case study development

In this study, we have used Yin’s approach to case study development [29], aiming to create descriptions of “typical survivorship pathways” to be further discussed with healthcare professionals and patient advocates in focus groups. To do so, we combined two sources of information – field notes from unstructured participant observations of follow-up appointments [30] and “survivorship stories” of paediatric cancer survivors and their informal caregivers, collected as part of episodic narrative interviews [31]. To consider challenges during transition, we also interviewed a group of survivors in a transition from paediatric to adult healthcare facilities. Following Yin’s approach, we ensured internal validity through triangulation of data sources (observations and narratives) and additional checking with healthcare professionals (TL). We addressed construct validity by operationally defining “survivor pathways” using established frameworks (Levesque et al.‘s care accessibility dimensions [32]). For external validity, we developed “typical” rather than unique cases, ensuring broader representativeness while acknowledging that analytical generalisation to theory (rather than statistical generalisation to populations) is the goal of case study research. Fig. 1 provides a general overview of the case study development.

We were aiming to develop two case studies to describe common, though not individual, experiences of survivors [33]. The decision to create two distinct cases reflects the fundamentally different challenges faced by adult survivors of paediatric cancer, who reached adulthood (in following – “adult survivors”), versus those in transition from paediatric to adult healthcare (in following – “transition survivors”). Adult survivors primarily deal with long-term late effects management and healthcare navigation autonomy, while transition patients face the additional complexity of changing healthcare systems during a vulnerable developmental period. This dual approach allows healthcare professionals in focus groups to discuss both established survivorship care (adult case) and the critical transition period (transition case), providing comprehensive insights for improving care across the survivorship continuum. For observations, transition patients were defined as survivors aged 16–25 attending appointments specifically designated as ‘transition visits’ or discussing transfer to adult care. For interviews, transition status was defined as survivors aged 18–25 who were currently transferring from pediatric to adult healthcare providers. We consider the “paediatric cancer survivor pathway” as a combination of physical, social, and psychological experiences framed by external factors of follow-up care accessibility. We used the care accessibility definition as suggested by Levesque et al., including the following dimensions [32]:

- Approachability, defined as a capability to effectively recognise the need for care (e.g. LTFU), and existing follow-up opportunities, and gain access to them;
- Acceptability, involving cultural and societal factors influencing a survivor’s willingness to embrace aspects of care, as well as how they evaluate the suitability of seeking care;
- Availability, encompassing the (physical) reachability of healthcare facilities with adequate capacity to deliver health services;
- Affordability, reflecting survivors’ and their informal caregivers’ economic ability to allocate resources in terms of both time and money to utilise appropriate services;
- Appropriateness signifies the alignment between services and survivors’ needs, including timeliness, thoroughness in assessing health issues and determining suitable treatment, and the technical and interpersonal quality of the services rendered.

The main aim was to describe a “typical journey” of a paediatric cancer survivor, with particular emphasis on organisational factors. These case studies were designed to capture survivors’ perspectives on how follow-up care is organised. We then used them to stimulate discussions in focus groups with healthcare professionals, highlighting how differing professional routines and disciplinary priorities can contribute to fragmented views on follow-up care [34].

### Data collection

Between June and November 2022, EA, possessing expertise in observations and psychological education but an outsider to the medical field, conducted 19 unstructured participant observations. The researcher approached the observation without preconceived notions, intending to explore real experiences during an appointment at a university hospital paediatric cancer follow-up centre. Before commencing the observation, EA signed a personal data statement. Additionally, written informed consent was obtained from all participating patients. The healthcare professional involved had limited prior knowledge of the study’s purpose to minimise any potential Hawthorne effect. Field notes were chosen over audio/video recording to capture non-verbal interactions and environmental factors while maintaining the confidential nature of medical appointments and reducing patient/provider anxiety about being recorded [35]. Being a healthcare professional, TL supervised the observation process, and before the analyses, field notes were discussed with him.

To recruit participants, we posted advertisements on specific social media platforms (such as the Facebook and Twitter accounts of the Children Cancer Foundation^1^) and in the magazine for paediatric cancer survivors “WIR”. We also collaborated with self-help groups and distributed study materials within the participating university hospitals. We adjusted the convenience sampling approach to obtain a heterogeneous group of participants concerning age, diagnoses, duration of follow-up, or grade of cancer survived [36]. To ensure the comparability of answers, participants with insufficient cognitive function were not included in the study. The eligibility criteria were:

### Observation eligibility criteria

Paediatric cancer survivors (cancer diagnosed before age 18) attending routine follow-up appointments at the university hospital paediatric cancer follow-up centre; ability to provide written informed consent; healthcare professional consent for observation; appointments conducted in the German language.

### Interview eligibility criteria

Paediatric cancer survivors diagnosed before age 18, currently aged ≥ 16 years, completed acute treatment, sufficient cognitive and language abilities for interview participation, written informed consent (with parental consent required for participants under the age of 18 years), willingness to discuss survivorship experiences.

Nineteen follow-up appointments with paediatric cancer survivors were observed, involving participants aged 12 years to over 35 years and representing a balance of male and female survivors. Diagnoses included both hematologic and solid tumours, with time since completion of acute therapy ranging from 5 to 30 years. Eight participants were classified as transition patients, and nearly half were accompanied by an informal caregiver. A general description of observed appointments is presented in Table 1.

**Table 1:** General description of observed appointments

	Sex	Age group	Diagnosis	Years since the end of acute therapy	Transition patient?	Informal caregiver?
1	F	35 +	Osteosarcoma	21	N	N
2	M	12–17	NHL	6	N	Y
3	F	18–25	Osteosarcoma	6	N	Y
4	M	35 +	HL	25	N	N
5	M	18–25	AML	6	Y	N
6	M	25–34	ALL	23	N	N
7	M	25–34	HL	20	N	N
8	F	18–25	NHL	14	N	Y
9	F	18–25	Ewing sarcoma	8	N	N
10	F	25–34	Medulloblastoma	29	N	N
11	M	18–25	ALL	5	Y	N
12	M	25–34	GCT	19	N	N
13	M	18–25	ALL	30	Y	N
14	F	18–25	Astrocytoma	6	Y	Y
15	F	12–17	AML	8	N	Y
16	M	18–25	Medulloblastoma	15	Y	Y
17	F	18–25	Ependymoma	15	Y	Y
18	F	18–25	NET	9	Y	N
19	M	18–25	NHL	7	Y	Y

Diagnosis:* HL* Hodgkin’s lymphoma, *NHL* Non-Hodgkin lymphoma, *AML* Acute myeloid leukemia, *ALL* Acute lymphoblastic leukemia, *GCT* Germ cell tumour, *NET* Neuroendocrine tumour, *Y *Yes, *N* No

In addition, 36 episodic narrative interviews in German language were conducted with a heterogeneous group of survivors and informal caregivers, covering a wide age range (born 1967–2010) and diverse diagnoses. The sample included both transition-age survivors and adults long past treatment, with varying follow-up durations, treatment experiences, and late-effect burdens, providing a rich basis for thematic analysis. 35 Interviews were conducted in person and 1 online due to the pandemic situation between September 2022 and March 2023. Interviews consisted of two different parts: personal narratives on the “survivorship story” from interview transcripts induced by a single open question and several blocks of questions with a more specific focus on themes induced from the theory of planned behaviour. The interviews were arranged in multiple centres across various regions of Germany, including University Hospital Schleswig-Holstein, University Hospital Bonn, and University Hospital Magdeburg. These centres encompass clinics with established structures for specialised multidisciplinary follow-up care after paediatric cancer and those without. Interview development is described elsewhere [28]. Written informed consent was obtained from all participants, for minor participants, the signature of a caregiver was also required. The general characteristics of interview participants are presented in Table 2.

**Table 2:** General characteristics of interviewees

	Year of birth	Diagnosis	Year of Diagnoses	Transition patient (18–25 years old)	Adult patient	Child	Informal caregiver	Relapse
1	1996	suprasellar germinoma	2016		X			N
2	1992	HL	2021		X			N
3	1993	Rhabdomyosarcoma	2004		X			N
4	2000	Bone cancer	2009	X				N
5	2006	Osteosarcoma	2018	X				N
6	2006	HL	2021	X				N
7	2005	Osteosarcoma	2020	X				N
8	2002	Ewing’s sarcoma	2018	X				N
9	2003	ALL	2016	X				N
10	2004	Nephroblastoma	2012	X				N
11	2006	Osteosarcoma	2018				X	N
12	2006	HL	2021				X	N
13	2005	Osteosarcoma	2020				X	N
14	2002	Ewing’s sarcoma	2018				X	N
15	2003	ALL	2016				X	N
16	2004	Nephroblastoma	2012				X	N
17	2001	Neuroblastoma	2019		X			N
18	1982	ALL	1985		X			N
19	2000	NET	2003		X			N
20	2003	Brain tumour	2016		X			N
21	2000	Brain tumour	2003		X			Y
22	1988	HL	2001		X			N
23	1967	Leukaemia	2005		X			Y
24	2004	Rhabdomyosarcoma	2008			X		N
25	2006	Neuroblastoma	2016			X		N
26	2003	Brain tumour	2016				X	N
27	2003	Brain tumour	2011				X	N
28	2004	Rhabdomyosarcoma	2008				X	N
29	2003	Neuroblastom	2017	X				Y
30	1978	HL	2005	X				N
31	2004	CML	2017	X				N
32	2010	ALL	2019			X		N
33	2008	ALL	2020			X		N
34	2004	CML	2017				X	N
35	2008	ALL	2020				X	N
36	2010	ALL	2019				X	N

Diagnosis: *HL* Hodgkin’s lymphoma, *NHL* Non-Hodgkin lymphoma, *CML* Chronic myeloid leukemia, *ALL* Acute lymphoblastic leukemia, *NET* Neuroendocrine tumor, *Y* Yes, *N* No

We conducted the study according to German Good Scientific Practice (“Gute Wissenschaftliche Praxis”) [37] in combination with the Good Epidemiology Practice and the Good Practice of Secondary Data Analysis for the whole VersKiK-Study [38, 39].

### Analyses

To generate topics, we analysed observation field notes and the personal narratives on the “survivorship story” from the transcribed interviews, based on Yin’s approach to generalisation for case studies in health services research [40], using specialised Software MaxQDA. To benefit from the full range of themes brought up by survivors and their informal caregivers, we used an inductive narrative approach to analyse the transcripts and field notes [41]. To do so, we applied Hsieh and Shannon’s five-step approach to data analysis [42]. These steps included: transforming text into narrative form, identifying units of analysis and themes, establishing coding rules, implementing the coding system across all narrative data and refining it as necessary, and finally, validating and selecting the ultimate dataset. Themes were defined by EA and reviewed by KB. We systematically categorised significant ideas and identified recurring themes within the narratives using thematic coding. To effectively capture the essence of the messages, we coded them based on the identified themes and subdivided them if needed. We reached inductive thematic saturation when no new topics emerged from additional observation field notes and interview narratives. This occurred after analysing 9 observations and 14 interviews, with the remaining data serving to confirm and enrich existing themes rather than generate new ones. We followed the principle that saturation in case study development occurs when additional data no longer contributes new insights to the emerging patterns, consistent with Yin’s recommendations for case study research [40].

## Results

We analysed narratives and field notes for adults and survivors in transition separately, organising the identified topics according to Levesque’s five dimensions of care accessibility (Table 3). This framework provided a systematic approach to understanding how survivors navigate follow-up care across different aspects of healthcare access.

**Table 3:** Description of survivors’ experience based on the levesque framework [43]

Dimensions of the Lavesque framework	Adult survivors	Survivors in the transition process and informal caregivers^a^
Approachability	Perception of survivors’ health, incl. emotional burden experience, and health-related self-efficacy	Health-related self-efficacy
Acceptability	Role of social environment, incl. autonomy	Emotional burden and insecurity Role of social environment in the transition, incl. perception and support of the environment, involvement of informal caregivers in the follow-up care
Availability	Organisation and structure of follow-up care, incl. the structural process of follow-up care, organisational obstacles and (additional) components of follow-up care offers like psychosocial counselling	Organisation and structure of follow-up care during the transition process, incl. the structural process of follow-up care, organisational obstacles and components of follow-up care offers
Affordability	Continuity of care, incl. familiarity with the healthcare environment and change of healthcare professionals in charge	Continuity of care during transition
Appropriateness	Patient empowerment, incl. transmitting information relevant to follow-up care and specifics of communication with healthcare professionals	Patient empowerment in the transition process, incl. transmitting information relevant to follow-up care and specifics of communication with healthcare professionals

^a^Informal caregivers are grouped with transition patients as their involvement is most prominent and complex during the transition period, when young adults are developing healthcare autonomy

### Adult survivors

Analysis of observation field notes from follow-up appointments in adults formerly diagnosed with paediatric cancer and interview narratives revealed five overarching topics that mapped onto Levesque’s dimensions:

#### Approachability: survivors’ perception of own health (HP)

Adult survivors demonstrated varying abilities to recognise their need for follow-up care and access available services. Many survivors expressed that regular check-ups provided psychological reassurance: *“So it was never the case that anything else was discovered*,* such as a secondary illness or a complication. But for peace of mind*,* initially mainly for my parents*,* but now I do it out of intrinsic motivation*,* it is somehow very important to come here to have everything checked out”* (Adult survivor). This category encompassed survivors’ experiences of emotional burden, anxiety, worries, as well as health-related self-efficacy in managing their long-term care needs. Statements about survivors’ perception of their health and independence in healthcare as well as the implementation of healthy lifestyle recommendations, are subsumed in this category.

#### Acceptability: role of the social environment (SE)

This dimension captured survivors’ willingness to seek care, influenced by cultural and social factors. Adult survivors frequently described the importance of social support while simultaneously striving for autonomy: *“I feel safer then. And*,* yes*,* I feel more secure when I have someone with me whom I trust… but it’s just that somehow*,* I feel better when there’s a second person there”* (Adult survivor). The category emphasised emotional and social support in follow-up care contexts and included statements about survivors’ developing autonomy from their informal caregivers.

#### Availability: organization and structure of follow-up care (SC)

Adult survivors faced significant challenges related to the physical accessibility and capacity of healthcare services. This was particularly evident in their descriptions of care coordination difficulties: *“Now I spend the whole year organising doctor’s appointments*,* getting check-ups and monitoring everything”* (Adult survivor). This category addressed healthcare system-related challenges, including difficulties finding knowledgeable doctors, obtaining timely appointments, and the need for regular contact with healthcare professionals who understand late effects of paediatric cancer.

#### Affordability: continuity of care (CC)

The economic and time-related aspects of accessing care were reflected in survivors’ experiences with care continuity. Several survivors reported that follow-up care involves additional costs not fully reimbursed by statutory health insurance. One explained: *“you had to go to the eye doctor*,* hearing aid provider*,* and so on”* (Adult survivor), highlighting services that typically result in out-of-pocket payments. Another described being *“busy the whole year organising doctor’s appointments*,* having everything checked in every area … I have diabetes*,* circulatory problems*,* fatigue and metabolic disorders”* (Adult survivor), illustrating the continuous need for specialist care beyond what insurance routinely covers. Moreover, frequent changes in healthcare providers created emotional costs: *“What can be said about the follow-up care for children? Since one doctor left*,* I’ve had a different one every time. And that’s always the case… there was simply no relationship of trust”* (Adult survivor). This category included familiarity with healthcare environments and the impact of changing responsible healthcare professionals, as well as survivors’ experiences when follow-up care was interrupted due to relocation, uncertainty, financial or time constraints.

#### Appropriateness: patient empowerment (PE)

This dimension addressed the alignment between services and survivors’ needs, particularly regarding communication quality and information transfer. Adult survivors often described inadequate communication: *“…that normal general practitioners are also relatively quickly overwhelmed… you could sense the uncertainty that a doctor doesn’t want to do anything wrong*,* which of course then leads to you not really feeling comfortable in your own skin”* (Adult survivor). The category encompassed both successful and unsuccessful aspects of doctor-patient communication, including handling contradictions between current and previous recommendations, active patient involvement, and aspects of shared decision-making.

### Survivors in a transition process

For survivors transitioning from paediatric to adult care, we identified six topics that reflected the additional complexity of this developmental period:

#### Approachability: health-related self-efficacy (HSE)

Transition survivors showed varying abilities to recognise care needs while managing developmental changes. One survivor described the security that regular monitoring provided: *“And I also think that it always gives you a certain sense of security when you know that*,* okay*,* at least the things that have been checked are fine*,* so that nothing needs to be done in terms of interventions or anything else”* (Transition survivor). This category included survivors’ perception of their health, healthcare autonomy, and implementation of healthy lifestyle recommendations.

#### Acceptability: emotional burden and insecurity (EB) and role of social environment in transition (SE)

The transition period was marked by heightened emotional challenges and complex family dynamics. Survivors described intense anxiety around medical examinations: *“So*,* the first MRIs were always at Grandma’s*,* Grandpa’s*,* Mum’s*,* always with a knot in my stomach*,* wondering whether there would be something to see or not. So*,* at the beginning*,* it was always hell*,* wondering whether there would be something to see*,* and then you get a call saying there’s nothing to see*,* and you’re relieved”* (Transition survivor). This category describes the role of emotional and social support either by informal caregivers or by the broader community in the context of follow-up care. Negative aspects of constant accompaniment (e.g., interference and overprotection) by informal caregivers are also reflected here.

At the same time, informal caregivers also experienced significant stress: *“We were*,* the closer the surgery date was*,* the shorter the time of follow-up care was*,* correspondingly very*,* very tense. Because many memories from the time before and from the surgery time just boiled up again and always resonated: ‘Hopefully everything is good’”* (Informal Caregiver). This dimension captured both the emotional challenges of transition and the complex role of family support versus developing independence.

#### Availability: organisation and structure of follow-up care during transition (CS)

Transition survivors faced particular challenges in accessing appropriate care services. The logistical complexities were evident: *“I have various doctors who have treated me. At the beginning*,* I had to go there very frequently. But I found it very good at the beginning that I was here or somewhere else almost every other week”* (Transition survivor). However, the transition to adult care often involved difficult decisions about care locations: *“That’s why he would have liked to stay in paediatric oncology*,* but of course that’s not possible at some point. Because of his age*,* of course. And Kiel then recommended us to come here to Luebeck*,* which is quite a long way for us. It takes us two hours to get here”* (Informal Caregiver). The organisation of the follow-up appointments, the adjustment/coordination of the examinations, and the efficiency of the processes are subsumed in this category. In addition, statements describing the search for information on follow-up care offers are marked in this category.

#### Affordability: continuity of care during transition (CCT)

The transition period created additional costs related to care continuity. Survivors expressed preferences for consistent providers: *“I would find it nicer if the same doctor was always there. If someone is always there who already knows what it’s about. And otherwise*,* I always have to tell again what’s going on*,* how I got here in the first place*,* stuff like that”* (Transition survivor). Caregivers emphasised the impersonal nature of fragmented care: *“Well*,* that’s it -- otherwise you’re just a case number. And then*,* when they always have to check first who’s sitting there? What’s in the file?”* (Informal Caregiver).

#### Appropriateness: patient empowerment in the transition process (PE)

Communication quality was particularly crucial during transition, with survivors valuing clear, comprehensive information: *“And what I always liked was that you could access it quickly… And what I find particularly beneficial is that you are informed of the values each time. Where they are located and what each value means. That there is a high level of communication”* (Transition survivor). This category was especially important for transition patients, encompassing both successful and unsuccessful communication experiences, including the significance of conveying examination results and health status understandably. This involves navigating contradictions between current and previous follow-up recommendations, active involvement of the patient, and aspects of shared decision-making or directive behaviour by the doctor. Additionally, the significance of conveying examination results and the state of health understandably is emphasised.

The topics identified through our analysis are systematically organised in Table 3 according to Levesque’s framework, with supporting quotes demonstrating how survivors’ experiences manifest across each dimension of care accessibility. This organisation reveals how adult survivors and transition survivors face distinct but overlapping challenges in navigating follow-up care, informing the development of our two case studies presented in Attachment 1. Case Study 1 was created to portray a standard scenario of an adult survivor, with a deliberate choice to focus on a female patient. This decision was motivated by the intention to explore various adult-life topics and to incorporate discussions about secondary neoplasms along with regular diagnostic procedures for preventive measures [44, 45]. The selection of the cancer type was based on its high prevalence [46] and the typical high burden of somatic late effects increasing over time, flanked by specific needs for psychosocial care.

In the second case study, we aimed to address challenges commonly faced by transition patients [47]. We opted to depict a male transition patient, considering the prevalence and frequency of the chosen diagnosis. Specifically, we chose a former brain tumour patient who had undergone a typical, intricate treatment regimen, resulting in inevitable late effects (e.g [48]).,, conflicting with striving for autonomy.

The case studies were constructed by selecting the most frequently mentioned topics from each survivor group and weaving them into coherent narratives. We have indicated the represented topics by implementing the topic abbreviations at the end of each paragraph. For example, Patient C’s experience with care interruption reflects narratives from twelve adult survivors who described similar follow-up gaps, while her anxiety about fertility represents concerns voiced by eight female survivors during interviews. Similarly, Patient M’s struggles with peer relationships and academic accommodations emerged from observations of five transition appointments and interviews with seven transition-age survivors. Case Study 1 (adult survivor) emphasised topics related to healthcare autonomy, continuity of care disruptions, and long-term health management, while Case Study 2 (transition patient) focused on themes of family involvement, educational challenges, and healthcare system navigation during developmental transitions. Case studies are presented in Attachment 1.

## Discussion

Enabling a thorough understanding of experiences and interactions bounded by a context, the case study approach is a suitable tool for studying complex phenomena in healthcare research [49, 50]. To create case studies, using the topics defined, we followed Wholeys’ “logic model” [51] as recommended by Yin [40], to capture “cause-effect-cause-effect-cause-effect” correlations. Wholey’s “logic model” provided the structural foundation for organising cause-and-effect relationships within our case studies, helping us trace how healthcare system factors lead to survivor experiences and outcomes. Levesque’s framework served as an analytical lens for categorising themes according to care accessibility dimensions. Aiming to gain an in-depth understanding of typical experiences made by both adult survivors and survivors during the transition, we designed two case studies, reflecting mostly discussed topics. Those topics could also be outlined utilising Levesque’s conceptual framework [43].

### Approachability

One of the main reasons for non-attendance of follow-up described in previous research is a lack of information, including insufficient knowledge about it, the perception that such care was unnecessary, and a diminished sense of urgency to visit a survivorship clinic due to their current state of subjective good health [52–54]. Additionally, it was shown that survivors and their informal caregivers may be lacking information on the type of cancer and its treatment [55]. Similarly, our results have shown that the availability of timely and sufficient information on the type of cancer and its therapy, their possible late effects, and follow-up opportunities affects survivorship experience both physiologically as well as psychologically – e.g. giving a feeling of “having one’s condition under control”. Health-related self-efficacy is generally shown to be positively associated with better survivorship experience and intention to attend follow-up [56]. Considering the limited availability of follow-up opportunities at specialised facilities (multidisciplinary follow-up appointments at university clinic) due to resource constraints currently in Germany, survivors and their informal caregivers typically search for support from ambulant specialists – e.g. GPs or paediatricians, - being dependent on their knowledge of late effects of paediatric cancer [57], requiring better collaboration between specialised paediatric oncologists and primary care specialists [58–60]. In addition, the need for regular contact with a healthcare professional with sufficient knowledge of late effects after childhood and adolescent cancer was also frequently recognised.

### Acceptability

Paediatric cancer survivors, influenced by potential traumatic experiences during the diagnostic and treatment phase, might develop aversions to clinical settings or experience fear of discovering late effects or relapse, negatively affecting readiness to attend follow-up [61]. These issues frequently emerged in narratives of survivors in transition and were observed at follow-up appointments with adult survivors with a higher comorbidity burden and greater need for LTFU care, including psychological counselling. Transition patients frequently discussed their psychological and practical preparedness for moving from familiar pediatric settings to adult healthcare environments, including them feeling safe with increased personal responsibility for healthcare decisions and their anxiety about leaving known providers (e.g [14]). A connected topic reflecting the role of the social environment (primarily family members as informal caregivers) was reflected twofold. Parents often perceive themselves as advocates and accompany transition patients and even adults to their follow-up appointments to engage them in follow-up care [62]. Another reason to join a follow-up appointment was similarly shown by Ginsberg et al. – a fear of handing over the responsibility to survivors themselves as a result of overprotectiveness [63]. The latter might cause problems with communication: as shown by previous research, paediatric cancer survivors want to be involved in the decision-making process, with an increasing desire to make such decisions alone or together with healthcare professionals and without family member involvement as the age increases [64]. Positive involvement of informal caregivers was also observed – in this case, they were either providing practical assistance [65] or serving as informational support. Secondly, friends and further “important others” form survivors’ social environment, playing an especially important role for survivors in the transition process.

### Availability

A limited number of specialized follow-up care providers is a significant factor affecting the survivorship pathway, as survivors and their informal caregivers have to face complex follow-up care self-management issues, including the necessity of organizing a significant number of appointments by different specialists, to collect complex medical documents by themselves, long traveling distances and different access to care needed in different regions of Germany, long waiting times, etc. These findings mirror those reported by Daly et al. [66] explaining the low engagement of a high proportion of paediatric cancer survivors in follow-up for similar reasons. Another topic discussed in both groups was continuity of care. Survivors and their informal caregivers expressed fear of a lack of familiarity and trust in the relationship previously established with paediatric oncologists [67]. Accordingly, the absence of a familiar paediatric environment was previously shown to be a special challenge for both survivors and their informal caregivers [43].

### Affordability

Another issue affecting survivorship experiences is cost. Consistent with previous research [54, 68], survivors and their informal caregivers articulated that they were unable to access the required follow-up care because their late effects were not recognised by insurance companies or they were not eligible for supplementary payments. Survivors also recognised that the absence of financial aid and the perceived high cost of medical expenses, including screening examinations, posed barriers to accessing survivorship care. Both patients and their informal caregivers described their limited capacity to meet often time-consuming and stressful care needs. This mirrors the difficulties outlined in the literature, demonstrating how young survivors are increasingly tasked with managing follow-up care among personal, social, and environmental challenges (e.g [69]).

### Appropriateness

As shown by previous research (e.g [43]). and frequently addressed topics in narratives and during observations, patient empowerment is crucial for better survivorship experiences and sufficient adherence to follow-up. Communication and information transfer between survivors and/or their informal caregivers and primary care providers, especially in the transition from paediatric to adult care, is mostly perceived as difficult. Considering current funding and structural gaps in follow-up care provision, encouraging health-related self-efficacy is essential to ensure that survivors possess the confidence and skills required to interact consciously and effectively with available healthcare services [61].

This study demonstrates the innovative potential of combining unstructured participant observations with episodic narrative interviews in survivorship research. This methodological triangulation captures both the objective dynamics of healthcare encounters and the subjective meaning-making processes of survivors, providing a more comprehensive understanding than either method alone. Our approach offers a replicable framework for developing evidence-based case studies that can inform healthcare professional training and policy development in survivorship care.

Combining a narrative approach with unstructured participant observations to develop a case, we were able to subsume relevant topics into concise survivorship pathways. Therefore, the main strength of the present study is the combination of two qualitative approaches. Unstructured participant observation allowed for an impartial understanding of follow-up care dynamics during follow-up appointments. Simultaneously, the analysis of survivors and their informal caregivers’ narratives provided a means to explore paediatric cancer survivors’ subjective needs and opinions, which ultimately contributes to triangulation in terms of research methods. Observation conducted by a non-healthcare professional added value to the study, as this approach facilitated the consideration of factors that may be overlooked in an interview-based study or by someone with healthcare expertise.

However, the study has several limitations. Since observation was conducted only on one site, we may not have captured the complete spectrum of survivors’ perspectives. However, personal narratives of survivors and their informal caregivers that were obtained as a part of a multi-centric episodic narrative interview may compensate for this difficulty. Having the obvious strength of free storytelling, - which increases the likelihood that experiences are recounted in rich detail, episodic narrative interviews evoke emotional responses. The resulting risks of selection, recall, and hindsight biases may be interpreted as limitations of this study. The voluntary basis of interview participation might also be referred to as a limitation of this study. The combination of inductive narrative analysis with case study development may introduce selection bias, as researchers inevitably make subjective decisions about which themes to emphasise in case construction. Additionally, the integration of observational and narrative data may privilege more articulate participants’ voices or more dramatic clinical encounters. We addressed these limitations through systematic member checking and by ensuring case studies represented common rather than exceptional experiences through focus group discussions. Apart from this, the episodic narrative interviews may have been influenced by interviewer bias, as EA’s psychological background and research interests could have unconsciously shaped question formulation or interpretation of responses. However, this was partially mitigated by EA’s outsider status to medical practice and by collaborative analysis with medical team members. Additionally, all observations and the majority of interviews were conducted in university clinics with an established organisational structure for multidisciplinary follow-up care. Thus, our results might not fully reveal the real situation faced by survivors receiving follow-up care outside such facilities – this perspective is only captured by previous experiences of interviewed survivors, fully leaving aside a perspective of survivors who are not involved in follow-up care.

## Conclusion

Continued LTFU care is a vital component for ensuring long-term survival after paediatric cancer, and its significance increases as survivors age. Consideration of survivors’ perspectives, needs, and preferences in the organisation of follow-up care can considerably improve adherence to follow-up recommendations, thus increasing survivors’ quality of life and quality of care. Case study creation to describe “typical survivors’ pathways” can provide insight into real-life experiences of cancer survivors and their informal caregivers, which might otherwise be overlooked due to the fragmentation of the healthcare system in general, thus giving a possibility to the improvement of LTFU care organisation.

Case studies development as a methodological approach might provide a better possibility for in-depth research of patients’ and informal caregivers’ experiences in an existing healthcare environment. Meanwhile, we used the case studies to carry out focus group discussions moderated by two typical scenarios (with up to eight participants each) within the VersKiK study, which were organised in Luebeck and Bonn [34]. Healthcare professionals involved in different stages of follow-up care who represent different institutions, including representatives of self-help groups and primary care providers, will come together to explore the level of knowledge of follow-up guidelines as well as current challenges regarding intersectional and interdisciplinary cooperation.

The case study development methodology demonstrated here has potential applications beyond the thematic field of (paediatric) cancer survivorship. This approach could be valuable for understanding patient pathways in other chronic conditions’ diagnoses, both during paediatric and adult age, and requiring long-term follow-up care, such as congenital heart disease, diabetes, or several mental health conditions. The combination of observational and narrative data provides a robust foundation for developing training materials and policy recommendations across various healthcare contexts requiring coordination between multiple providers and transitions between different healthcare settings.

## Supplementary Information

Supplementary Material 1.

## Data Availability

The anonymised datasets analysed during the current study are available from the corresponding author on reasonable request under consideration of the Ethic Committee’s Votum and European General Date Protection Regulation.

## Footnotes

1 Deutsche Kinderkrebsstiftung - Gemeinsam für krebskranke Kinder.
