# Supplementary material for: Case study in VersKiK: a methodological approach for studying paediatric cancer survivors’ pathways
Source: BMC Med Res Methodol. 2025 Dec 2;25:274. doi: 10.1186/s12874-025-02723-x (PMC12696934; doi:10.1186/s12874-025-02723-x)
Supplement: Supplementary file 1 — Supplementary Material 1. [file 12874_2025_2723_MOESM1_ESM.docx]

**Attachment 1**

**Case Study 1.**

**Adult woman aged between 30 and 40 years;**

**Diagnosed with lymph node cancer at age 10;**

**Treated with both chemo- and radiotherapy, and has undergone surgery;**

**20 years since end of acute treatment;**

**Focus of follow-up care: prevention**

Patient C, 35 years old, was diagnosed with lymph node cancer 20 years ago. Her comprehensive treatment included chemotherapy, radiation, and surgery. After successful acute therapy in 2003 and completion of protocol-based follow-up care, she is now in long-term follow-up. Regular appointments and check-ups provided her with a sense of security, as she knew an experienced multidisciplinary team ***(CC)*** was caring her***.***

Over time, the follow-up intervals were extended to six month, as no signs of recurrence were detected ***(SC).*** Despite stable results, Patient C sometimes felt insecure and anxious about the follow-up examinations ***(HP).*** The fear of possible recurrence of the disease and the uncertainty about her future health, especially the question of whether she would be able to have children with her partner, emotionally burdened her (HP). Work and family commitment often clashed with follow-up appointments ***(SC).*** Organising doctors’ appointments and managing follow-up examinations required additional time and planning, leading to stress ***(SC/HP)***.

These challenges were worsen by frequent relocations due to her partner’s career changes ***(SC).*** Patient C even interrupted her follow-up care for a few years between the ages of 25 and 30 - despite a formally successful transition to adult medicine ***(SC).*** However, her cousin eventually encouraged her to schedule a follow-up appointment after reading a newspaper article about the importance of lifelong follow-up care ***(SE).*** She even briefly considered asking her cousin to accompany her to the appointment, especially given her concerns about possible fertility treatments being too late, and the fear of discussing this issue with her partner ***(SE/HP).***

The lack of continuity in care affected her trust in medical care and led to uncertainties about the quality of follow-up ***(CC/HP).*** Patient C sometimes felt abandoned and wished for more information about possible late effects of her treatments ***(HP/PE).*** Sometimes she had the impression that the doctors simply wanted to inform her quickly about certain topics but did not even consider whether she was receptive to them at that moment ***(PE).*** She also felt that during the discussions her anxieties about each new examination were not sufficiently taken into account ***(PE/HP).*** She missed support and clear information on the importance of prevention and the role of a healthy lifestyle in long-term follow-up care ***(PE).*** She would have been happy to discuss this, as well as her changes in appearance after the therapy, with other affected women of her age ***(SE/PE).*** Difficulties also arose in finding a doctor with sufficient knowledge of late effects after paediatric cancer ***(SC/CC).***

In order to cope with these anxieties, she would have liked to have psychological support within her follow-up. However, she had the overall impression that she had coped well with the serious illness and felt even often more mature than her fellow students did when it came to coping with daily challenges and demands during her studies and when starting her career ***(SC/SE).***

Despite these difficulties, patient C emphasises the importance of prevention and continuous follow-up examinations ***(PE).*** She recognises that follow-up care is crucial to detect and treat possible relapses or late effects early on ***(PE).***

**Case Study 2.**

**Young man aged between 16 and 18 years;**

**Diagnosed with a brain tumor at age 6;**

**Treated with chemo- and radiotherapy, and has undergone neurosurgery;**

**Has a hearing aid;**

**8 years since end of acute therapy;**

**Focus of follow-up: treatment of late effects**

Patient M, a young man now aged 18, was diagnosed with a brain tumour eight years ago. He underwent challenging treatment including chemo- and radiotherapy as well as neurosurgery. The therapy was successful but it also left long-term side effects, including hearing loss that requires him to wear a hearing aid. Since the end of the acute therapy, Patient M is participating in regular appointments in follow-up care. Before he turned 18, he was told to change to the adult health care facilities and continue his follow-up appointments there ***(SC/CCT).***

One topic that is often addressed during follow-up appointments is his hearing aid, since it is physically and emotionally challenging him to get used to it in everyday life ***(EB/HSE/PE).*** Outside his follow-up appointments, he feels like he lacks support regarding his difficulties that come with having a hearing device ***(SC).*** Another area that is affected by his former illness is his growth, thus he needs to receive regular hormonal treatment. His mother often helps him to inject hormones, as this is a complicated procedure for him ***(HSE/SE/PE).*** Therefore, he needs to stay home often, to receive the dosage in time ***(HSE/SE).*** An endocrinologist regularly checks the hormone levels ***(SC/HSE).*** Additionally, a neuropsychological examination needs to be done to specify his cognitive impairments by a psychologist, and he is currently waiting to get an appointment ***(SC).***

Due to all of his restrictions and special needs Patient M. and his family often feel overwhelmed ***(EB).*** He needs to find a way to psychologically cope with his impairments and find a way to integrate it into his life ***(EB/HSE/PE).*** Patient M. and his family also need to adapt to the new situation that he is becoming a grown-up and needs to make more medical decisions on his own ***(SE/HSE).*** He notices that he is starting to feel uncomfortable when his mother is present during follow-up appointments that concern his future like having children or choosing a career ***(EB/SE/HSE).*** This is especially hard for his mother, since she still supports him a lot ***(SE/PE).*** Worries and anxieties about a relapse or appearance of a new tumour are constantly present ***(EB).***

Patient M. also suffers from learning and concentration difficulties at school, because of his brain tumour. It was brought up to him to make use of handicap compensation, e.g. more time during exams, but as he didn’t wanted to be considered as “weak” by his classmates it wasn’t implied yet **(SC/SE/EB).** Moreover, because of his long treatment, he has spent a lot of time in the hospital, and has lost contact with many of his friends, which sometimes makes him feel isolated ***(SE/EB).*** It is hard for him now to interact with others, due to his hearing impairments and changes in physical appearance ***(EB/SE/HSE).*** He needs support to feel comfortable again in different social environments ***(EB/SE/HSE).*** However, Max regularly attends a sports group in which young people with and without disabilities play sports together ***(HSE/SE).*** He likes the fact that now, after COVID-19, these appointments are now regularly take place again after Corona and that there are two exercise instructors who lead different indoor and outdoor activities ***(HSE/SE).*** In general, it is easier for him to get involved in leisure activities if the situations are pre-structured by others ***(SE/PE).***

Generally, his follow-up care requires continuous monitoring and care by different specialists ***(SC/CCT).*** For him and his family it is challenging to organise the necessary appointments and examinations and to balance the time needed for these with other activities, such as school and leisure activities and his mothers' working hours ***(SC/PE).*** In addition, the change to adult health care facilities is quite challenging, as they have to find the right specialists ***(SC/CCT/PE/HSE/SE).*** For the new doctors they need to collect and bring all patient records and it feels burdening to repeat the medical history over and over ***(SC/PE/HSE/SE/CCT).*** On top, they experience a lack of time during new appointments and have the feeling that they lack sufficient explanations ***(SC/CCT/EB).*** Max quickly gets angry when he does not understand something and then withdraws from the conversation altogether because he has the impression of being patronised ***(PE/SC/EB/HSE).*** At the same time, this withdrawal reinforces his dependence on his mother and he feels even more patronised, creating a vicious cycle ***(SE/HSE/PE/EB/SC).***
